# Dental Infection and Resistance—Global Health Consequences

**DOI:** 10.3390/dj7010022

**Published:** 2019-03-01

**Authors:** Mainul Haque, Massimo Sartelli, Seraj Zohurul Haque

**Affiliations:** 1 Unit of Pharmacology, Faculty of Medicine and Defence Health, Universiti Pertahanan Nasional Malaysia (National Defence University of Malaysia), Kem Sungai Besi, 57000 Kuala Lumpur, Malaysia; 2 Department of Surgery, Macerata Hospital, via Santa Lucia 2, 62100 Macerata, Italy; massimosartelli@infectionsinsurgery.org; 3 Ninewells Hospital & Medical School, Dundee DD1 9SY, Scotland, UK; szzhaque@gmail.com

**Keywords:** dental, oral, maxillofacial, infection, antibiotic, antimicrobial, resistance pattern, epidemiology, common microorganism, nutrition component, biofilm formation, morbidity, mortality, increase healthcare cost

## Abstract

Antibiotics are widely used in dental caries and another dental related issues, both for therapeutic and prophylactic reasons. Unfortunately, in recent years the use of antibiotics has been accompanied by the rapid emergence antimicrobial resistance. Dental caries and periodontal diseases are historically known as the top oral health burden in both developing and developed nations affecting around 20–50% of the population of this planet and the uppermost reason for tooth loss. Dental surgeons and family practitioners frequently prescribed antimicrobials for their patients as outpatient care. Several studies reported that antibiotics are often irrationally- and overprescribed in dental diseases which is the basis of antimicrobial resistance. The aim of this review is to evaluate the use of antibiotics in dental diseases. Almost certainly the promotion of primary oral health care (POHC) in primary health care program especially among the least and middle-income countries (LMIC) may be the answer to ensure and promote rational dental care.

## 1. Introduction

Penicillin was the first antimicrobial agent discovered in 1928 [1] and the first patient was treated with this newly discovered medicine in 1942 [2]. The therapeutic potential was soon well recognized. World War II promoted extraordinary cooperation between two nations situated either side of the Atlantic, United States and Great Britain, resulting in unbelievably efficient bulk production of penicillin by 1943 [1,3]. Dutch scientists during post World War II developed a new method of producing penicillin which was marketed in 1946, and ultimately ensured mass production of penicillin for the medicine market and reduced the cost of treatment [3,4]. Antibiotics have transformed modern medicine and countless lives have been saved through their use over the years. The invention antibiotic was a turning point for medical science and the quality of human healthcare. Unfortunately, the use of this miracle medicine has been accompanied by the rapid emergence of resistant strains due to the unstoppable spread of antibiotic resistance genes among the microbial community [5,6]. The microbial community develop defense strategies for their existence against the antimicrobial assault, which is a prime instance of microbiological metamorphosis, transformation, and the zenith of evolution. “An immense genetic plasticity of bacterial pathogens that trigger specific responses that result in mutational adaptations, acquisition of genetic material or alteration of gene expression producing resistance to virtually all antibiotics currently available in clinical practice” is considered as the natural law [7]. In the early 1950s, Japan experienced an epidemic of Shigellosis caused by antibiotic resistance strains of *Shigella.* A few years later Japanese scientists described that resistance to “multiple antibiotics not only developed quickly and simultaneously, but also seemed to transfer from resistant to sensitive strains” [8]. Additionally, before penicillin was marketed for treatment, a bacterial penicillinase (β-lactamases) was recognized by Abraham and Chain, two members of the penicillin invention squad [9]. It is interesting that microbes developed their existence technology, such as β-lactamases, before antibiotics were ever used. A recent database voiced the presence of more than 20,000 possible resistance genes (r genes) of strictly 400 different types, as analyzed from available bacterial genome sequences [10,11]. Microorganism resistances to antimicrobials now extend to all known categories of agents, either natural or synthetic. Antimicrobial resistance (AMR) has not only stalled treatment approaches to infectious diseases but has also influenced healthcare policy and planning [10]. Subsequently, medical specialists are facing the possibility that human beings may return to the pre-antibiotic era of medical care [5] as AMR is threatening not only effective treatment towards bacterial diseases but also other microbes such as parasites, viruses, and fungi [12,13]. There were a lot of reports that dental surgeons often prescribed large amounts of antimicrobials. Often these antibiotics were overprescribed, or prescribed imprudently and irrationally, which in turn promotes microbial resistance. This manuscript will try to discuss issues related to dental infection and resistance and its impact on global health consequences. 

## 2. Materials and Methods

This review has been based on freely accessible literature from Google, Google Scholar, and PubMed, and from the link provided by the Universiti Pertahanan Nasional Malaysia ((UPNM) National Defence University of Malaysia). The terms used were dental, oral, maxillofacial infection, resistance pattern, epidemiology, common microorganism, nutrition component, biofilm formation, morbidity, mortality, and increase healthcare cost. A few manuscripts incorporated required payment to view the full paper but have been provided free of charge by other libraries as part of cooperation with UPNM. This is a narrative review article will give effort to describe and deliberate issues related to dental infection and resistance and its impact on global health significances, from a theoretical and contextual point of view, based on previously published manuscript. Additionally, there has been no attempt to utilize specific a data base and to use a methodological approach to develop a systematic review and meta-analysis. 

## 3. Epidemiology of Dental Infection

Dental caries and periodontal diseases are historically known as the top oral health burden in both developing and developed nations affecting around 20–50% of the population of this planet, and is the uppermost reason for tooth loss [14,15,16]. Among Indian patients over 30 years of age it has been reported that the significant (almost 80%) cause of loss of teeth is due to periodontal disease [17,18]. Multiple studies reported that in Asia, the Middle East, and across the African sub-Saharan regions, dental caries is a principal public oral health threat [19,20,21]. Though some studies state that globally dental caries has declined in the population especially in modern countries [21,22,23], other recent research reports that there is a significant increase in rates of caries [24,25].

Another Indian study said that periodontal disease is widespread in the Eastern Indian state of West Bengal [26]. One Libyan study conducted among a population of 1225 subjects aged 18–34 years, revealed that only 5% population had healthy periodontium [27]. Multiple studies reported that periodontal disease occurrence and severity increases with patients’ age [28,29,30,31]. Additionally, periodontal diseases occur more often in males and individuals with poor oral hygiene practice [32,33,34]. Pediatric and adult groups of males were more often sufferers of dental infections and other infections of the oral cavity in comparison with the female population of both rural and urban communities [35,36,37]. One Dutch study revealed that the incidence of caries decreased in the period of 1990–2009 among an 8–21-year-old studied population in both low and high socioeconomic cluster and was also statistically significant [38]. Similarly, another British study conducted among 69,318 children aged 5–15 years revealed a 31–51% reduction of caries in the last 40 years (1973–2013). This study also reported a greater reduction among 15-years age group. Additionally, the British study concluded that although there is a significant improvement, caries continues to be a significant liability to the national healthcare system [39]. In Norway, prevalence of caries decreased from 81% to 52.2% in a 15-year period (1985–2000) among 12-year-old children. However, the next 4 years (2001–2004) the prevalence increased to 59.8% with a 3.3% annual growth rate. Nevertheless, the decayed, missing, and filled teeth (DMFT) index kept on steady around 1.6 from 1997 to 2004 [40]. A similar DMFT was also observed in other Scandinavian countries [41]. The DMFT index has been in use by dental surgeons for nearly 80 years and persists as the most reliable tool to assess dental caries for epidemiological study [42]. Another research among 28,522 German first grade children revealed that 25.9% had caries and required treatment. This study examined in further detail 25,020 children and reported that girls possess better oral and dental health when compared to boys regarding decayed missing and filled primary teeth (DMFT). There was a statistically significant (*p* < 0.001) difference between DMFT score among sexes. Boys DMFT score was double than that of girls [43].

## 4. Common Microorganisms Involved in Dental Infection

Periodontal disease and dental caries are the most frequently reported chronic infective dental diseases and are caused by microorganisms living in the mouth cavity [44,45]. The human oral flora encompasses over 700 microorganisms, and 50% of those microorganisms are uncultivable microbes [45]. The different anatomic areas and its bathing fluid, saliva of the oral cavity, possess oral microbiome comprising the normal oral flora which includes bacteria, archaea, fungi, protozoa, and viruses. These oral microbiomes usually swim in saliva as free-floating microorganisms and form a complex ecological community of biofilm, attaching to different surfaces of the mouth cavity [46,47]. Biofilm is often responsible for several local and systemic diseases [47].

One Japanese study reported that diverse infection of strict anaerobes with facultative anaerobes, especially commensal streptococcal gram-positive bacteria known as viridans streptococci, was identified as the principal pathogens responsible for dentoalveolar infections, periodontitis, and pericoronitis [48]. Another Japanese study identified *Fusobacterium*, *Peptostreptococcus micros*, *Porphyromonas* species, and *Prevotella* species as culprits for dentoalveolar infection [49]. Additionally, 34% of Prevotella species were found to produce beta-lactamase [49]. *Peptostreptococcus micros* was also identified by another group of researchers as responsible for progressive periodontitis [50]. Multiple studies revealed polymicrobial flora with frequent involvement of Gram-negative anaerobic pathogens [51,52,53,54,55,56]. Similar other studies reported that 90% of root canal infections were due to obligatory anaerobic microbes [52,57].

## 5. Nutrition Component That Promotes Dental Infection

Diet also plays a substantial role in dental caries and enamel erosion [20,58,59]. “Caries is demineralization of the inorganic part of the tooth with the dissolution of the organic substance due to a multifactorial etiology” [60]. Other researchers define “dental caries is a multifactorial disease that results from interactions among a susceptive host, caries-related bacteria, and cariogenic diets” [61]. The demineralization of the dental enamel and dentine by organic acids that form in the dental plaque are because of anaerobic microorganisms metabolizing sugars of the diet [62]. Dietary machinery especially dietary acids contribute to the development of enamel defects (e.g., enamel hypoplasia, fluorosis) [58]. Soft drinks contain both acids and sugars, have the acidogenic and cariogenic property that can lead to dental caries and enamel erosion. Several research studies revealed a positive correlation between drinking soft drinks and dental caries and erosion [63,64,65]. One study conducted among children of 2–10 years revealed that those children who consumed a high volume of carbonated soft drink also had a significantly higher rate of dental caries than children who had high juice, high milk, and high water in their diet [66]. Additionally, one British study reported that boys of 14–15 years take much more sugary drinks than that of their female counterpart. Two or more glasses of sugary drink have a statistically significant association with dental caries. The high consumption of sugary drink was found to have a correlation with family income, gender, and mother’s education level [67]. Another American study suggests that present-day high consumption of fizzy drink among children, above all the escalation in “soda pop” drinking, were responsible for an intensification of dental caries rates [68]. This study and other research additionally revealed that poor intakes of micronutrients such as riboflavin, copper, vitamin D, and vitamin B_12_ are associated with an increased rate of caries and gingival diseases [68,69]. Low intake of milk and other dairy products has been associated with carries among children [68]. Other comparable studies also similarly condemned that high intake of non-alcoholic carbonated drinks and low consumption of milk and other dairy products among children and adolescents increases the risk for dental caries and other systemic diseases [63,70,71]. Multiple studies reported that drinking milk ensures the consumption of many macronutrients and micronutrients which in turn promotes and protects health from various diseases [72,73,74]. Very low-level sugar consumption was observed among the communities of Inuit of Alaska, Ethiopia, Ghana, Nigeria, Sudan, and the islands of Tristan da Cunha and Sant’Elena. These populations often lead traditional lifestyles and rate of carries were found to be very negligible. Researchers observed that as the economic condition of these geographical areas improve, the amount of sugar and other fermentable carbohydrate content increases in the diet, and this paralleled the increases rate of caries [62,75,76,77]. Additional studies revealed that frequent consumption of fermentable sugars promotes caries formation [78,79], as sugar such as sucrose when fermented to produce lactic acid which lowers pH which ultimately disturbs the environment and damages normal the demineralization and remineralization process, consequentially results in dental enamel destruction through demineralization [78,80].

## 6. Biofilm Formation among Dental Infection

Researchers with the aid of advanced technology have identified a rich microbial consortium and around 1000 diverse microbial species, which include bacteria, virus, and fungi in the dental biofilm. Biofilms are a structurally and functionally organized community of microorganisms with a three-dimensional configuration and are surrounded in a matrix of extracellular material [81,82,83]. Among microbes existing in the oral biofilm, many of them are innocent natural inhabitants. Nevertheless, some of them have the inherent quality of damaging the mineralized and soft tissues of teeth and gum [83]. Antonie Philips van Leeuwenhoek, a Dutch scientist in the seventeenth century, first reported regarding observed dental plaque and oral biofilms, and plaque is responsible for caries, and periodontal disease [84]. Multiple studies reported that more than 10^11^ microorganisms per mg of dental plaque were found [45,85,86]. Several studies reported that the mouth cavity is rich in nutrition, temperature 35/36 °C, pH 6.75–7.25 are the factors which all encourage the growth of many microorganisms and the formation of biofilm [87,88,89]. Just after tooth brushing and rinsing, the mouth cavity salivary proteins form a thin film over the tooth surfaces. Microorganisms with their microfilaments attach to this thin protein film. If the oral environment alters to become favorable for microorganisms, their population increases in number and communicates by secreting signal molecules and creates a community [90,91]. “The microbes secrete proteins, polysaccharides, nucleic acids, and other substances to the extracellular matrix, additionally containing proteins and nutrients from saliva” [83]. Thereafter, forms the matrix of the biofilm [81].

The principal microbial species found in the plaque of diseased areas are dissimilar from those of healthy areas. This is even though at the healthy locations of the mouth cavity, disease producing microbes are still regularly spotted but in lower numbers [92]. The former suppositions regarding the cause of the dental caries were believed to be due to the increased number of microorganisms in plaque “(Non-specific Plaque Hypothesis),” or that plaque contains particular types of cariogenic bacteria, such as *Streptococcus mutans* and *Streptococcus sobrinus*, within the plaque “(Specific Plaque Hypothesis) [85,93,94]. At present, the fundamental concepts of the earlier two hypotheses were reunited and widely accepted as the “Ecological Plaque Hypothesis.” The current hypothesis of caries formation describes that the normal oral microbial environment, if stressed, alters towards certain disease-related micro-organisms [61,95,96,97].

## 7. Antibiotic Use and Resistance Pattern of Microorganism in Dental Infections

The appearance of penicillin in the market and clinical use has saved many lives throughout the planet, transformed medical science and its’ success in treating infectious disease [3,98,99]. Poor use of antimicrobials promotes the emergence of antibiotic-resistant microbial strains [100,101], increases the possibility of antibiotic-associated adverse reactions [102], and characterizes a waste of healthcare funds [103]. Antimicrobial resistance is a grave public health and patient care issue. Antimicrobial resistant infections are exceedingly problematic in regards to the treatment of infectious diseases. Consequently, prudent use of antimicrobials demands the highest priority to prevent antimicrobial resistance, ensure patient safety and improve healthcare [103]. Multiple studies reported that dental surgeons frequently prescribed inappropriate antibiotics which ultimately promote antimicrobial resistance [103,104,105,106].

Antibiotics are widely used in dental caries and another dental related issues, both for therapeutic and prophylactic reasons [107]. Dental surgeons frequently prescribe antibiotics with apprehension that oral cavity normally contains huge number microorganism as normal flora which can cause infections in their patients [108]. It has been reported that antibiotic prophylaxis within 2 hours of dental procedures were beneficial [109], however, antibiotics taken after 4 hours had no benefit achieved [110]. Procedures such as periodontal surgery, scaling and professional teeth cleaning can cause significant bleeding from hard or soft tissues, and such bleeding cannot always be anticipated for prior to the procedure [110]. Another group of scientists believe that “indications for the use of systemic antibiotics in dentistry are limited since most dental and periodontal diseases are best managed by operative intervention and oral hygiene measures” [111]. Currently enormous amounts of antibiotics are prescribed and consumed because of dental infective disorders around the planet [106,112,113]. Regular and frequent use of antibiotics in dental infection often causes long-term public health troubles by leading to the development of resistant microbes including multidrug-resistant pathogens. This may be all the more tragic if there was minimal benefit to patient care by giving the antibiotic in these circumstances [114,115].

One study conducted in India comprising of 68 patients (18–58 years old) examined microbial specimens and reported that “a total of 64 aerobic and 87 anaerobic strains were isolated. The predominant bacteria were *Streptococci viridans* (64%), *Prevotella* (43%), *Peptostreptococcus* (26%), *Porphyromonas* (7%), and *Fusobacterium* (14%). The anaerobic Gram-negative bacilli (40%) were the predominant organisms followed by aerobic gram-positive cocci (34%). Four strains of *Candia albicans* were also identified” [116]. *Streptococci viridans* were highly sensitive to amoxicillin-clavulanate (95%), amoxicillin (90%), and levofloxacin (83%). Similarly, *Prevotella* were highly susceptible to amoxicillin-clavulanate (97%) and less sensitive to erythromycin (62%). Again, *Peptostreptococcus* and *Porphyromonas* are highly sensitive to amoxicillin-clavulanate (100%) and clindamycin (100%) [116]. The identified microorganisms were extremely sensitive to the commonly prescribed antimicrobials, e.g., amoxicillin + clavulanate combination and amoxicillin alone, clindamycin, and levofloxacin, but were more resistant towards erythromycin [116]. Another British study reported that an acute dental abscess was typically polymicrobial, with facultative anaerobes, such as viridans group streptococci and the *Streptococcus anginosus* cluster, with largely strict anaerobes, such as anaerobic cocci, *Prevotella* and *Fusobacterium* species. The highly sophisticated technology, based on non-culture techniques, identified a diverse community of pathogens, e.g., *Treponema* species and anaerobic Gram-positive rods such as *Bulleidia extructa*, *Cryptobacterium curtum,* and *Mogibacterium timidum* [117]. Macrolide antimicrobials were documented as less sensitive towards the ‘viridans group streptococci,’ anaerobic streptococci, *Streptococcus oralis*, and *Prevotella* species [48,118,119,120]. Another study reported that penicillin shows less reduced susceptibility towards the mitis streptococci group than the anginosus group [121]. The Infection Research Group of Glasgow Dental Hospital and School studied among 155 viridans group streptococci for their “minimum inhibitory concentrations (MICs) to penicillin, amoxicillin, ceftriaxone, erythromycin, clindamycin, rifampicin, vancomycin, and teicoplanin” and revealed that “27% of S. oralis were resistant to penicillin, 51% resistant to erythromycin, and 6% resistant to clindamycin. Further, 11% of S. mitis were resistant to penicillin, 40% resistant to erythromycin and 3% resistant to clindamycin.” Penicillin-resistant pathogens were also shown to be less sensitive towards other antimicrobials [122]. Researchers of the Barkatullah University, India studied gram-positive cocci (e.g., Streptococcus mutans, *S. sobrinus*, *S. oralis*, *S. sanguinis*) and bacilli (*Lactobacillus acidophilus*, *L. rhamnosus*, *L. fermentum*). These pathogens show resistance for each antibiotic in this research as follows: “penicillin V: 72/150 (48%), tetracycline: 99/150 (66%), amoxicillin: 135/150 (90%), cloxacillin: 117/150 (78%), and erythromycin: 90/150 (60%) [123].” Another study from Nepal identified that 91% of invading pathogens were gram-positive and 9% were gram negative. *Streptococcus mutans* were resistant to penicillin (66.15%), tetracycline (60.76%), and cotrimoxazole (20%). *S. aureus* was found to be resistant towards penicillin (91.48%), tetracycline (86.17%) and ampicillin (61.70%). *S. mitis* was resistant to tetracycline (78.12%) and ciprofloxacin (65.62%). *Pseudomonas spp*. were 100% resistant to tetracycline and cotrimoxazole was 90.90% [124]. Janaki medical school researchers reported that from bacterial isolates, 90% were Gram-positive. Among that Gram-positive microorganisms were *S. mutans, S. aureus*, *S. mitis*, *S. albums*, and *S. vestibularis*. The remaining 8.48% were Gram-negative microbial isolates. Again among Gram-negative: *Pseudomonas spp.*, *K. pneumoniae*, *P. vulgaris*, and *Enterobacter spp*. Among microbiological isolates were sucrose fermenter and non-sucrose fermenter 93.84% and 6.15% respectively. Documented gram-positive microorganisms were sensitive towards ciprofloxacin, gentamicin, and erythromycin 94.27%, 51.85%, 49.49% respectively. The gram-negative organisms was sensitive to ciprofloxacin, imipenem and gentamicin, and ceftriaxone 100%, 89.28%, and 50% respectively [125]. Another study from Brazil revealed that oral pathogens were highly resistant to ampicillin, amoxicillin+clavulanic acid, cefoxitin, cephalothin, amikacin, chloramphenicol, and nalidixic acid. This study also reported that carbapenems (meropenem and imipenem) were the most active antimicrobial agents and 1.6–2.3% pathogens showed resistance toward these medicine. Low resistance profile was also observed with ciprofloxacin and rifampin [126]. A Mexican study conducted among 60 children with active infections in the primary dentition revealed that Clindamycin in 8 lg/mL and 16 lg/mL exhibited the maximum (85.9%) microbial resistance followed by amoxicillin (43.7%) and amoxicillin-clavulanic acid (12.0%). *Streptococcus oralis* and *Prevotella intermedia*; *Treponema denticola* and *Porphyromonas gingivalis*; *Streptococcus mutans*; *Campylobacter rectus*; and *Streptococcus salivarius* were found 75%, 48.3%, 45.0%, and 40% respectively to be the most predominantly resistant microorganisms identified through polymerase chain reaction [127].

A composite microbial community exists normally in the mouth cavity which contains of diverse bacterial and fungal classes, their accompanying biofilms, and often with produce cytokine which promote relentless inflammation. These various infectious consequences of poor oral health among different age group of patients [92,128]. The Mayo Clinic reported that oral health had potential to enhance several diseases process which include endocarditis, cardiovascular diseases, pregnancy and birth, diabetes mellitus, HIV/AIDS, osteoporosis, Alzheimer’s disease, eating disorders, rheumatoid arthritis, head and neck cancers, and Sjogren’s syndrome [129]. It has been recommended that prescribing practice especially regarding antimicrobials need to be improved among dental surgeons and all health professionals, additionally, dentals surgeons were advised to follow guidelines for prescribing, and awareness regarding antibiotic resistance need build up among common people [114,130].

## 8. Morbidity, Mortality, and Increase Healthcare Cost in Dental Infections

Oral health is essential component of general health and quality of life. Thereafter, the World Health Organization (WHO) defines “It is a state of being free from the mouth and facial pain, oral and throat cancer, oral infection and sores, periodontal (gum) disease, tooth decay, tooth loss, and other diseases and disorders that limit an individual’s capacity in biting, chewing, smiling, speaking, and psychosocial well-being [131]”.

“Detrimental effects of oral infections on general health have been known for almost 3000 years” [132]. Chronic oral and dental disease can be very dangerous, especially among immunocompromised patients [131]. Consequently, different studies have suggested that such chronic infection promotes several life-threatening systemic diseases and increases morbidity and mortality [133,134,135,136,137]. Tooth loss impairs mastication and leads to higher mortality by causing poor diet, nutrition, and eating behavior [138,139,140]. One Japanese study, utilizing the multivariate-adjusted Cox proportional hazards model, revealed that practicing proper oral care, like that of tooth brushing, regular consultation with dental surgeons, and use of dentures, had an inverse relation with mortality among the elderly population [140,141]. Additionally, periodontal diseases and tooth loss are frequently related to coronary heart disease, stroke, pneumonia, and incident disability [142,143,144,145,146]. 

Chronic oral infections, particularly periodontitis, are the underlying cause of several fatal systemic diseases like endocarditis [86,147]. Several studies have shown that cardiovascular diseases such as coronary heart disease (CHD), stroke, peripheral vascular disease (PVD), cardiomyopathy, atherosclerosis, and myocardial infarction are linked to chronic infection and inflammation [148,149,150,151,152,153]. It has been shown that severe chronic microbial dental infections, endocarditis and meningitis are associated with cerebral infractions among male patients [154,155]. Additionally, research studies revealed that periodontitis has a potential role in regulating many other systemic diseases such as diabetes, respiratory disease, low birth weight, pre-term infants, other pregnancy-related issues, rheumatoid arthritis, and osteoporosis [156,157]. “Several mechanisms have been proposed to explain or support such theories, and oral lesions are indicators of disease progression, and oral cavity can be a window to overall health and body systems [156].” Chronic periodontitis is responsible for producing C-reactive proteins (CRP), interleukin-1b (1L-1b), interleukin-6 (1L-6), and Tumor Necrosis Factor-alpha (TNF-α) and disseminate these inflammatory mediator substances through the human body [158,159]. One Indian study revealed that patients with periodontitis had a higher level of high-sensitive (hs)-CRP than those who were suffering from gingivitis and with healthy gingiva [160]. The Mayo Clinic reported that “a high-sensitivity C-reactive protein (hs-CRP) test, which is more sensitive than a standard test, also can be used to evaluate your risk of developing coronary artery disease, a condition in which the arteries of your heart are narrowed. Coronary artery disease can lead to a heart attack [161].” Furthermore, high level of inflammatory mediator CRP increased the possibility of the development of type 2 diabetes mellitus (T2DM) and suggested that CRP is considered as a strong independent forecaster [162,163,164,165]. One recent study of Thailand reported that CRP, IL6, and TNF-α related with the initiation process for the development T2DM. This study additionally revealed that there was a strong co-relationship between CRP and T2DM. Consequently, researchers suggested that CRP is a good biomarker for the diagnosis and assessment of T2DM [166]. Similarly, one meta-analysis comprising of 19 manuscripts involving 39,136 participants and 7924 cases reported that “T2DM risk was strongly associated with elevated levels of inflammatory cytokines (IL-1β, IL-6, IL-18, CRP), TNF-α, and low levels of adiponectin [167]”. Another research studied three important inflammatory mediators (IL-6, CRP, and TNF-α) and revealed that among the elderly population, having low levels of these inflammatory meditators were linked with low death rate and improved quality of life [168]. Multiple studies reported that elevated CRP level indicate chronic periodontal disease, and other oral mucosal diseases including denture related edentulous lesions [169,170]. 

Chronic periodontal diseases often serve as a reservoir site to produce inflammatory mediators. These mediators have the potential to encourage threat to the fetal-placental unit and increase the risk of adverse pregnancy outcomes [171]. One Iranian study reported that mothers delivering low birth babies (LBW) had statistically significantly higher level of diseased gingiva (*p* = 0.042), and more deep pockets (*p* = 0.0006, Mann–Whitney test) [171]. This study concluded that chronic periodontal infections are one of the possible independent risk factors for LBW [171]. Similarly, one systematic review comprising of 12 prospective studies reported that a statistically significant risk of preterm delivery for those women suffering from periodontitis (risk ratio (RR):1.70) during pregnancy and a significant risk for LBW (RR: 2.11) [172]. Another similar systematic review and meta-analysis of case-control studies, comprising of 17 case-control studies and involving of a total of 10,148 patients, assessed periodontal disease as a risk factor for preterm birth, LBW babies. This study reported that “the estimated odds ratio was 1.78 (CI 95%: 1.58, 2.01) for preterm birth, 1.82 (CI 95%: 1.51, 1.20) for LBW and 3.00 (CI 95%: 1.93, 4.68) for preterm low birth-weight [173].” Another meta-analysis and systematic review involved 10 case-controlled studies from seven countries (India, Brazil, Iran, Argentina, Jordan, Senegal, and Tanzania) with a total of 2423 patient with a mean age ranging from 13–49 years [174]. This research meta-analysis also found that chronic periodontal infection among pregnant mothers often resulted in the delivery of a child before term and of LBW [174]. One more Indian case-control study concluded that pregnant women having chronic periodontal infections is a significant obstetric risk factor for preterm LBW. These patients of Nellore District, Andhra Pradesh, India frequently delivered preterm LBW child. This study finally commented that both antenatal status and periodontal diseases contribute to delivering preterm LBW child [175]. Age, sex, socioeconomic status, education level, race, genetics issues, CRP also had relation with dental diseases [28]. 

Dental diseases often remain untreated even in the USA and endure as a substantial public health threat. It has been advocated that preventive dental care improves overall oral health, but not all patients of the USA receive regular dental healthcare [176,177,178,179,180]. Over 10,000 patients visit five hospital systems (a total of seven hospitals) in the Minneapolis—St. Paul metropolitan area in Minnesota, USA for emergency dental care and it costs closely $5 million per year [175]. These patients were principally treated by minimizing their acute illness such as pain but not addressing underlying pathology of the disease [180]. Another study revealed that in the USA around 35% population or 100 million had no dental coverage [181]. One study reported that pediatric patients receiving treatment for dental diseases in a hospital operating room were the US $1508 and $104 for hospital non-operating room [182].

One Indian study reported that dental infections are a major public health threat, having a considerable influence on the quality of life, and work performance both academic and professional [178]. This study additionally reported that an enormous amount of monetary support is required to treat caries-related issues among school children of India. Nonetheless, the Indian budget for public health care is considerably under-resourced for oral health care [183]. Furthermore, in the Indian budget there is no precise, distinct provision for oral health care [183,184]. The healthcare in Malaysia is principally provided under a publicly operated system by the ministry of health which is almost free or has a very nominal charge at point of use [185]. Private health care is also available in big cities [185]. The government of Malaysia believes that “health is a public service to be made available to everyone, with equity of access, both in geographical and cost terms. The Ministry of Health is also the lead agency in the provision of oral healthcare to the nation” [186]. Oral healthcare in Malaysia is provided both by the public and private sectors [186]. 

On average Brazilian people spend R$42.19 annually on dental care which comprises dental insurance, appointments, and treatments. Again, R$5.10 is spent on dental insurance of the total dental care expenditure and of this amount R$4.70 was for private dental coverage. Nonetheless, only 2.5% of Brazilians spend money on dental insurance [187]. Another study reported that in Brazil dental care is traditionally based on private dental service [188].

Numerous peoples are suffering diverse issues of oral and dental diseases around the planet especially in countries having resource constraints, where government oral health care programs are limited [189]. Thereafter, the encumbrance oral diseases are very high all over the world against a low availability of skilled manpower regarding the management of oral health care [190]. At present multiple studies evidenced that there is urgent need to develop primary oral health care (POHC) programs all over the planet, especially in developing countries to promote effective oral health education and regular dental health check-up programs [189,190,191].

## 9. Primary Health Care and Primary Oral Health Care

Health is an ultimate human right which is requisite for the implementing of other issues human rights. Human rights are constitutionally preserved and ensured by almost all countries of this planet for persons and clusters against activities that restrict with their basic self-determinations and human rights. Thereafter, human rights are universal [192,193]. Primary health care ensure both health care and human rights both in developing and developed countries [194,195]. Currently, essential oral health service, regular dental care programs, and oral health education programs with intervention were found lacking throughout the globe especially in LMIC [196,197,198]. As POHC is an integral part of primary health care [191]. Thereafter, countries need developed POHC program to address this global issue of dental care [191].

## 10. Conclusions

The mouth cavity is the natural territory for various microbes. These naturally occurred microorganisms often act as a reservoir for resource for pathogenic microbes to produce local oral/dental infections and later systemic infections [199]. Antibiotics are often prescribed and used unnecessarily and excessively, which in turn contributes to the development of resistant microbes [200,201,202,203]. Multiple studies reported that at least 30–50% of antimicrobials were prescribed without any scientific reasoning [204,205]. Dental surgeons and family practitioners frequently prescribed antibiotics for their patients (24%) as outpatient care [206]. Several studies reported that antibiotics are often overprescribed by dental surgeons for non-indicated diseases and irrationally in dental diseases which is the basis of antimicrobial resistance [207,208,209]. Moreover, dental surgeons very rarely documented infection through performing culture-sensitivity tests and the majority of antimicrobial prescribing was the result of “guess work” and described as imprudent use [111,210]. Multiple earlier research studies advocated that dental surgeons should prescribe antibiotics/antimicrobials only to control documented, local systemic infections, but not for mere inflammation [111,211,212]. Additionally, prophylactic use of antimicrobials in dental disease was suggested for only for suspected systemic infective conditions [213,214]. 

Antimicrobial resistance is a natural phenomenon that occurs as microbes evolve. However, human activities have accelerated the pace at which microorganisms develop and disseminate resistance. Incorrect and inappropriate use of antibiotics are contributing to the development of such resistance. It is necessary that all healthcare workers must recognize dental infections play a central role in preventing the emergence and spread of resistance. The inclusion of POHC as a component of primary health care may be the answer to promote rational dental care. 
